# Variability of Microcystin-LR Standards Available from Seven Commercial Vendors

**DOI:** 10.3390/toxins14100705

**Published:** 2022-10-14

**Authors:** Donna Hill, Johnsie Lang, James McCord, Mark Strynar, Charlita Rosal, Judith Schmid, Thanh-Thao Le, Neil Chernoff

**Affiliations:** 1 Center for Public Health and Environmental Assessment, United States Environmental Protection Agency, Research Triangle Park, NC 27711, USA; 2 Arcadis (United States), 4204 Technology Dr, Durham, NC 27704, USA; 3 Center for Environmental Measurement and Modeling, United States Environmental Protection Agency, Research Triangle Park, NC 27711, USA; 4 Independent Researcher, Las Vegas, NV 89146, USA; 5 Almac Group, Durham, NC 27704, USA; 6 Independent Researcher, Raleigh, NC 27612, USA

**Keywords:** microcystin-LR, commercial cyanotoxin standard, mass accuracy, purity

## Abstract

Microcystins (MCs) are a large group of heptapeptide cyanobacterial toxins commonly produced in harmful algal blooms (HABs) and associated with adverse health effects in wildlife, livestock, pets, and humans. MC chemical standards are extracted from cyanobacteria biomass rather than produced synthetically and are used in water assessment methods and toxicological studies. MC standards are generally supplied in less than 1 mg quantities, and verification of the mass can only be accomplished by analytical chemistry methods using a certified reference of the specific MC for comparison. Analytical quantification of MCs in environmental samples and toxicology studies using accurate doses of test chemicals administered to experimental animals rely on the availability and accuracy of chemical standards. To check the accuracy and purity of available standards, seven individual microcystin-LR (MCLR) standards were purchased from separate commercial vendors and analyzed to determine the actual mass supplied and identify the presence of potential contaminants. To determine the effect of varying toxin mass in toxicological studies, each MCLR standard was administered to CD-1 mice in doses based on mass purchased, by a single 40 µg/kg intraperitoneal injection. The measured mass purchased varied from the vendor label mass by more than 35% for two of the seven MCLR standards. Contaminants, including trifluoroacetic acid (TFA), were identified in four of the seven samples. Comparative in vivo hepatotoxicity between vendor samples closely reflected the actual amount of MCLR present in each standard and demonstrated the toxicological impact of varying cyanotoxin mass.

## 1. Introduction

Microcystins (MCs) are a class of cyclic heptapeptides with more than 279 derivatives reported [1,2], produced by cyanobacteria (blue-green algae) in freshwater bodies throughout the world [3,4,5]. MCs have demonstrated hepatotoxicity in rodents [6,7,8,9,10,11] and an association with deaths of wild animals [12,13] as well as domestic pets [14,15,16] and livestock [17,18]. Deaths associated with incidental exposure to MCs are usually due to acute, high-dose exposures that inhibit protein phosphatases 1A and 2A in the liver which result in loss of cellular structure, cell death, and ultimately intrahepatic hemorrhage and liver dysfunction [19,20]. Potential human exposure routes have been reported as contact and ingestion of contaminated recreational water [21,22,23,24], drinking water [25,26], fish and shellfish [27,28,29], irrigated crops [30,31], and algal dietary supplements [32,33].

MCs are found worldwide and are the most common freshwater cyanotoxins in U.S. waters [34,35,36]. Quantitative monitoring of MCs in impacted waterways [37,38], as well as toxicological studies of microcystin-LR (MCLR) [6,7,8,9,10,11,39] and related compounds are dependent on access to high-purity and mass-accurate standards. Standards are typically extracted from cyanobacteria biomass as opposed to being synthetically prepared [40] and require validation to confirm the purity of extracted material and the accuracy of measured standard concentrations. A frequently cited extraction and purification procedure for MCs involves bulk extraction from dried algal cells with 5% acetic acid, gross separation with preparatory silica gel chromatography and high pressure liquid chromatography (HPLC) on a C18 column to isolate individual MC species [41]. MC standards prepared from these extractions are typically sold in milligram quantities in glass vials with HPLC-UV generally used for purity verification, which could lack accuracy in the quantity provided and limited identification of potential contaminants [25].

The concentration of MCs in aqueous environmental or laboratory samples can be determined using various analytical methods including enzyme linked immunosorbent assay (ELISA), ultraviolet–visible spectroscopy (UV-Vis) or liquid chromatography-mass spectrometry (LC-MS) [42,43]. Each method has benefits and drawbacks for MC analysis. While UV-Vis is relatively inexpensive and easy to use, it is not compound specific and has high detection limits. LC-MS allows for identification and quantitation of individual MCs, but often costs more compared to UV-Vis analysis. LC-MS analysis for MCs is typically performed using a triple quadrupole mass spectrometer. Using a mass spectrometer with a higher mass resolution, like a time of flight (TOF) mass spectrometer, allows for quantification of known MCs, but also identification of non-targeted compounds present in samples [44].

In monitoring environmental samples for MCLR and other MC congeners [37,38], inaccurate standard concentrations could produce widely varying results. The lack of standardization for quantity of commercially supplied MCs could confound method cross-validation and national efforts to coordinate investigations of MC-containing algal blooms. Most suppliers provide purity statements with their standards based on HPLC-Photodiode-Array Detection (PDA), but do not provide any verification that the quantity supplied is accurate. Since standards are supplied in quantities too small to weigh, end users are not able to verify the MC mass before use. Additionally, varying quantities for standards used in toxicological studies may invalidate reported dosage levels related to health effects. Standards may also contain impurities due to incomplete purification, or introduction of salts during the purification process. These contaminants could impact toxicological endpoints resulting in inaccurately reported health effects. For risk assessment of cyanotoxins, it is essential that the concentrations and purity are accurate.

Fastner [45] reported concentration measurements from 31 labs for one MCLR sample measured using the same reference material and found concentrations varied by 24–49% across labs. This previous study was designed to evaluate inter-laboratory variations in analytical measurements, not variabilities in standards provided from commercial vendors. Fastner does note that “about half of the participants found differences between quantification with the standard (provided) and their in-house MCLR standard by HPLC-PDA/UV (data not shown)”. The in-house standards in this study were a mix of commercially prepared and house prepared. A recent project report from The Water Research Foundation [43] included an interlaboratory comparison of 85 cyanotoxin standards from nine vendors which included certified reference materials (CRMs) and non-CRM standards. Differences were found in concentration and purity between vendors, but also between lots from the same vendor. Multiple cyanotoxin standards were evaluated including MCLR standard measurements which varied as much as 50%.

MCLR standards at the time for this study could be obtained from nine commercial vendors, but two of the vendors sold exclusively certified reference materials (CRMs). CRMs are sold as small volume methanol solutions verified by mass spectrometry, nuclear magnetic resonance (NMR), liquid chromatograph ultraviolet absorption (LC-UV), and liquid chromatograph chemiluminescence nitrogen detection (LC-CLND) and are used as the gold standard for quantity validation [46]. Using CRMs for toxicology studies would be cost prohibitive and require extensive handling to remove the methanol and combine multiple small amounts which would likely contribute to toxin losses. Studies requiring larger milligram (mg) quantities of MCLR would generally use dry standards. Dry MCLR standards were purchased from the remaining seven vendors although one vendor provided no information about sample purity (Supplemental Table S1). Five vendors used HPLC to verify purity, with two of these vendors using additional PDA testing to verify the delivered mass.

Our goal was to quantify the mass of MCLR purchased from seven different commercial vendors using three analytical methods performed independently and evaluate for impurities using a time-of-flight mass spectrometer (MS-TOF). To evaluate potential impact on toxicological effects, each MCLR standard was administered to CD-1 mice at 40 µg/kg in a single dose intraperitoneal (i.p.) injection based on the mass stated by the supplier and comparative toxicity was assessed using a 24 h observation, necropsy, and serum clinical chemistry.

A table with specific information about each vendor is included in Supplemental Materials (Table S1 Supplemental). Information labeled “certificate of analysis” (COA) was included with 4/7 vendor standards (C, D, F, and G) and an additional COA was available when requested (B). Of the five COAs, only 2 included chromatograms (D and F). Vendors A and E supplied a MSDS. Vendor A was the only sample without a lot number and a purity statement. Three vendors supplied a stability or expiration date (D, E, and F). Vendor E does not recommend aqueous storage for more than 24 h. The cost/mg ranged from $400 to $670.

## 2. Results

### 2.1. Chemical Analysis of MCLR Standards

**Figure 1:**
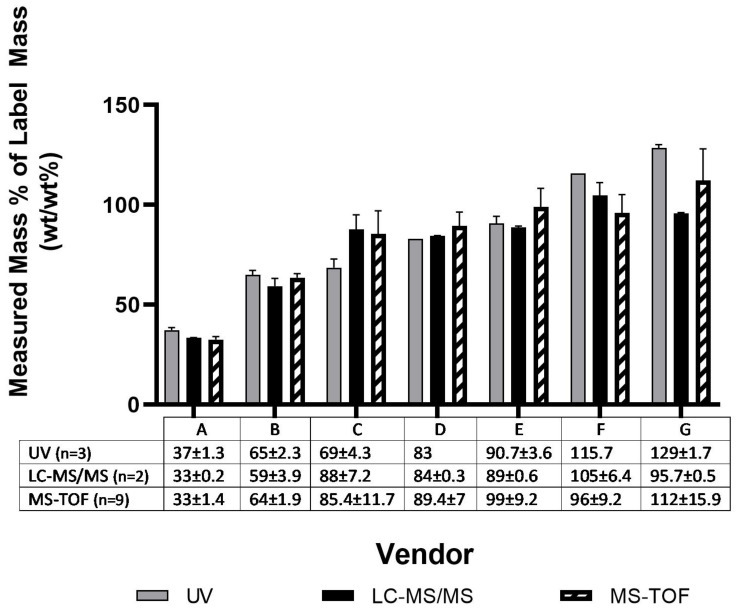
Quantity of MCLR measured compared to purchased quantity from seven different vendors in 2016, measured using UV, LC-MS/MS, and MS-TOF absorbance/Beer Lambert’s Law. Error bars represent one standard deviation for the average of each measurement technique.

Quantification of microcystin standards by multiple methods revealed that the majority (5/7) of the sources deviated from the reported concentration within ±20% regardless of measurement technique, with a smaller subset (2/7) deviating substantially (>35%) (Figure 1). Previous work has demonstrated losses of MCLR over multiple pipetting steps when using polypropylene pipette tips [47] which could result in systematic underreporting of concentrations. Sample preparation was consistent across vendors apart from Vendor B, where additional transfer steps were required to reconstitute the sample to the desired concentration. The additional transfer steps required to move sample from the purchased vial for Vendor B could, at least in part, account for the lower quantified concentration (Figure 1). Unintentionally, Vendor B also sat at room temperature in the original shipping package for seven days while all other vendor samples were placed in −20 °C storage upon arrival.

While many of the results demonstrated low variability in the sample means across the three analytical methods, Vendor G and Vendor C concentrations varied significantly (*p* < 0.05) between UV and LC-MS/MS methods (Figure 1). Discrepancies in MCLR concentration for a sample quantified with UV compared to LC-MS/MS suggests the presence of a contaminant in the sample that interferes (±) with light absorbance at 238 nm.

The MS-TOF non-targeted screening revealed 4/7 MCLR samples contained compounds that were not present in the sample blanks (Table 1). These four samples have the ratio of the contaminant peak area to the MCLR peak area reported here. No compounds other than MCLR were identified in the samples from Vendors C, D and G using the current methods. Negative mode screening revealed sulfate salt clusters in the Vendor E sample, with a peak area lower than the MCLR peak area. In positive mode, relatively small amounts of de-methylated MCLR and MCLR methyl-ester were identified in the Vendor B sample. Demethylated MCLR was the most common contaminant found in the Guo study [43], but in most cases was less than 5% and the authors concluded that this amount was not likely to affect quantification. The identity of these MCLR variants were verified with monoisotopic mass and retention time comparison to the National Research Council Canada (NRC Canada) CRM containing documented, low levels of the MCLR variants.

Trifluoroacetic acid (TFA) was present in the samples from Vendor A and F, with peak areas 11 and 18 times the MCLR peak for these samples, respectively (Table 1). Peak area ratios should not be used to infer relative concentrations of contaminants because of variable levels of molecular ionization in the mass spectrometer. The TFA in samples from Vendors A and F matched the monoisotopic mass and retention time of a TFA standard (Agilent Technologies) analyzed on the MS-TOF following the identification of the 113.993 monoisotopic mass in the vendor samples. The presence of TFA (i.e., acid buffer) could have improved the dissolution of the MCLR in the sample vials. Through communication with vendors, it was discovered that the Vendor A standard had been measured against a CRM that was diluted to half-strength. The vendor measuring the standard was not aware of this at the time.

### 2.2. Toxicological Comparison of MCLR Standards

**Figure 2:**
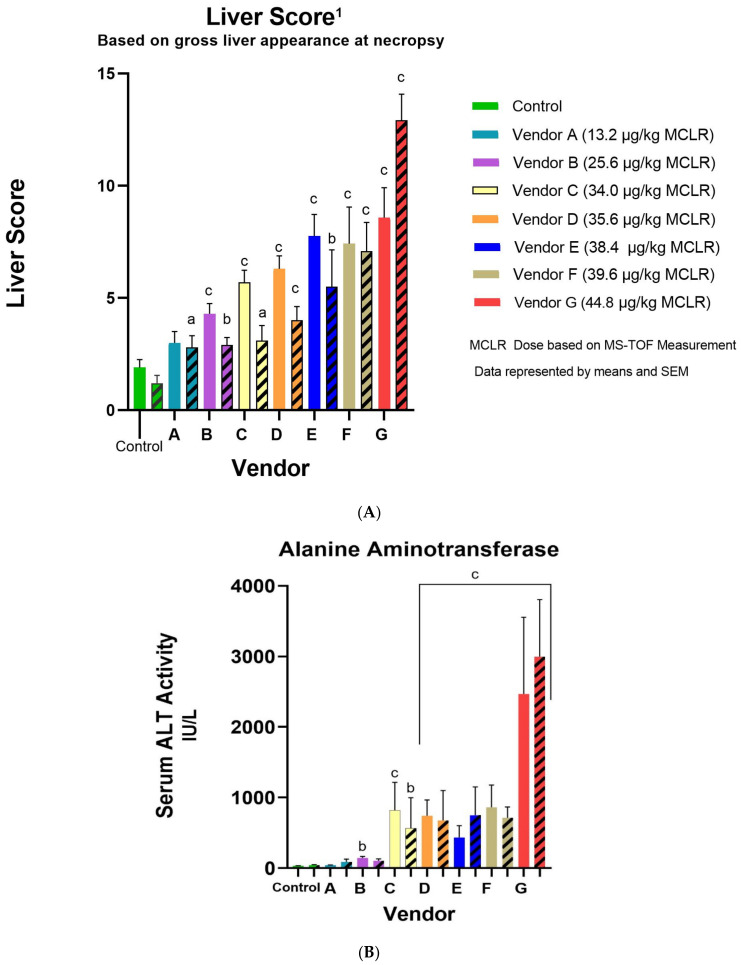

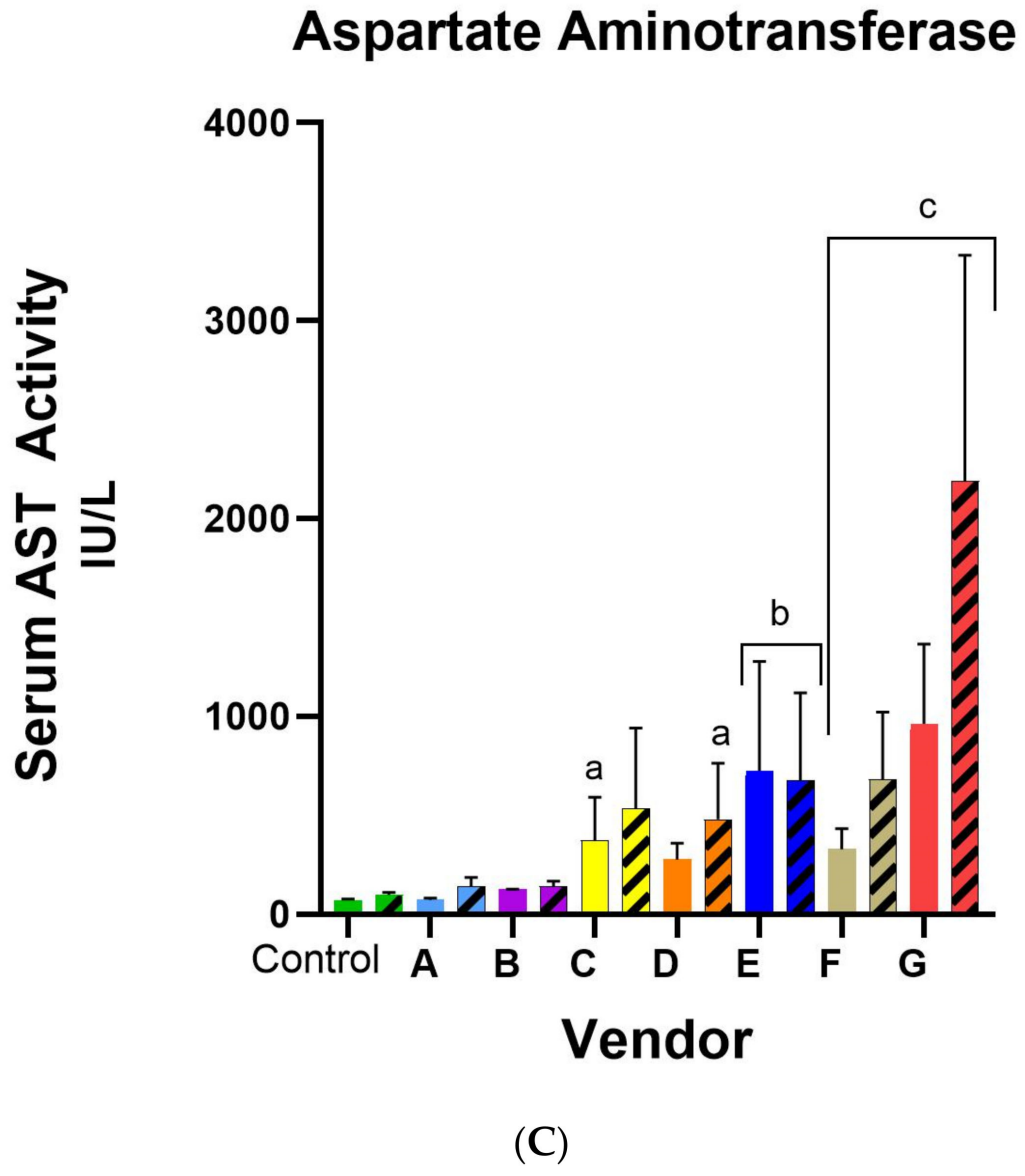
Graphs comparing mouse liver score (**A**), ALT (**B**), and AST (**C**) among the seven vendors. ^1^ Liver Score is a gross assessment of the liver appearance at necropsy and is assigned a value based on the presence and extent of hemorrhage, white infiltration (lipid or glycogen), and reticulation on liver outer surface. Assignment of normal liver score = 0–2, mild = 3–5, moderate = 6–8, severe = 9–15, death = 16 (euthanasia < 12 h post-dosing). MCLR percentages are based on the MS-TOF results. Males are represented by solid colors and females have the diagonal lines. 1 Alanine aminotransferase (ALT) is an enzyme which leaks from injured hepatocytes’ cytoplasm. Aspartate aminotransferase (AST) is another enzyme found in hepatocytes (and other cell types) that leaks extracellularly into the blood when the liver is injured. Males are represented by solid colors and females have the diagonal lines. a = *p* < 0.05, b = *p* < 0.01, c = *p* < 0.001.

Microcystin-LR (MCLR) is a commonly occurring and commonly studied hepatotoxic cyanotoxin and is used here to demonstrate the importance of accurate cyanotoxin stock concentration in toxicology studies. MCLR exhibits a steep dose–response [39] which results in measurable effects from small changes in dose. Because of this, the differences in hepatotoxicity endpoints in vivo generally mirror the variations in MCLR standard concentrations (results in Table 2). The liver score (defined in Section 4.2.2), which assesses the gross appearance of the liver at the time of necropsy [10,11], consistently increased with MCLR dose concentrations and reached significance with males in vendor groups B-G (MCLR dose ≥ 25.6 µg/kg) and females in vendor groups A-G (MCLR dose ≥ 13.2 µg/kg) compared to the controls (Figure 2A). The liver/body weight is used as opposed to liver weight alone to normalize for varying body weights. The liver/body weight increased in male Vendor F and G (38.4 and 44.8 µg/kg MCLR) while only to significance in male Vendor G, but the female liver/body weight increased with increasing MCLR dose and reached significance in Vendors C-G (MCLR dose ≥ 34 µg/kg). Other strong associations were seen in increasing serum markers for hepatic injury with alanine aminotransferase (ALT) significantly increased for males in vendor groups B-G and females C-G (MCLR dose ≥ 34 µg/kg) (Figure 2B). Aspartate aminotransferase (AST), another enzyme associated with hepatic damage, increased with MCLR exposure, and reached significance for males in vendor groups C (MCLR dose = 34 µg/kg), and E-G (MCLR dose ≥ 38.4 µg/kg) and females in groups D-G (MCLR dose ≥ 35.6 µg/kg) (Figure 2C). Glutamate dehydrogenase (GLDH), a hepatic mitochondrial enzyme released when hepatocytes are damaged, was also significantly increased in both sexes for groups B-G (MCLR dose ≥ 25.6 µg/kg). There was no GLDH data for males in Vendor F due to a lab equipment malfunction.

In this study, the non-hepatic markers were less related to the MCLR dose. The markers for renal function had sporadic significant decreases in blood urea nitrogen (BUN) for male Vendors A and D, female Vendors A and C; decrease creatinine (Cr) in male Vendor B and female Vendors A and F. While increases of BUN and Cr are usually the changes indicating kidney disease, true decreases are less common and can reflect severe muscle atrophy, liver disease, or protein starvation [48,49]. The BUN and Cr decreases in these mice were not correlated to MCLR concentration. Starvation and muscle wasting are conditions requiring chronicity and were not present in this 24 h study. It is likely that the controls had values on the higher end of normal which caused the comparison to appear as decreases in the treated groups. The females in vendor group G did show a highly significant increase in BUN (*p* ≤ 0.001) and paired with the control range creatinine is often reflective of dehydration and is possible in this group with the highest concentration of MCLR. Vendor G females did have the highest liver/BWT ratio with only a mean weight loss of 0.24 g. Liver weight increase from blood accumulation could hide larger BWT decreases that would be expected with dehydration.

Blood proteins were measured as albumin, which is made entirely in the liver, globulins which are immune-response proteins partially made in the liver, and total protein which is the summation of the albumin and globulins [50,51]. Males had increased albumin in vendor group E, decreased albumin in vendor group F while the females in vendor group G had a striking decrease (*p* ≤ 0.001). Vendor group G females also had a decrease in globulins while females in vendor groups B and D and males in vendor groups C, E, and G had increases in globulins. The only significant change in total proteins was an increase in the Vendor F males (*p* ≤ 0.05). Changes that can occur within a 24 h timeframe, as in this study, would be mild dehydration, inflammation, and early liver dysfunction. Dehydration can cause increases in all three protein parameters, inflammation can increase globulins, and liver dysfunction could potentially cause decreases in all three parameters. Overall, albumin tended to be stable or increase, but not to significance, in groups with the lower concentrations of MCLR and decrease in the groups with the higher concentrations of MCLR (with the exception listed above for a significant decrease in males in Vendor E).

Early deaths are defined as mice requiring euthanasia before the 24 h post-dosing timepoint due to animal welfare issues (hypothermia, severe lethargy, pale ears indicating anemia). Vendors E, F and G had early deaths, both sexes, totaling three, four and nine, respectively.

## 3. Discussion

In this study, MCLR standards were purchased from seven individual commercial vendors and first analyzed for quantity and purity using UV-Vis, LC-MS/MS and MS-TOF. The actual quantity of the standards was within 20% of the label quantity for 5/7, while 2/7 differed by >35% from the value reported on the vendor’s label. Due to several factors inherent in the process for performing mass spectrometry, ±20% of the target value is usually considered an acceptable range [37,52].

A non-targeted analysis using the MS-TOF showed contaminants in 4/7 standards, two of which appeared to have high levels of trifluoroacetic acid (TFA). As mentioned above, the comparative mass of the contaminants cannot be determined from the ratios (due to ionization differences) to calculate the purity percentage which all, but Vendor A assured was >95% by HPLC or HPLC-PDA. Of the four vendors with the highest amount of MCLR, all four had COAs, but only two included chromatograms of the purity analysis. The cost of the top four also included the lowest and highest price per mg. A conversation with a customer representative for Vendor G revealed that it is their policy to include an extra 10% of product per container although this information was not available on the label or on accompanying documentation.

Each of the seven vendors’ standards were used as dosing solutions to demonstrate the effect of variable MCLR concentrations on a toxicological evaluation. CD-1 mice received the dosing solutions based on the MCLR quantity indicated by the vendor as a single i.p. injection dose of 40 µg/kg. The 40 µg/kg dose level was selected from a pilot dose–response study in preparation for this study (Supplemental Figure S1). MCLR is a hepatotoxicant with a steep dose–response [39] and these characteristics helped elucidate a range of severity in liver toxicity endpoints that generally mirrored each vendor sample MCLR concentration. Vendors E, F, and G had the highest MCLR concentration ranging from 89–129% of the label mass (35.6–51.6 µg/kg MCLR). The percent deaths for these three vendors, respectively were 13%, 17% and 38% which strengthens the evidence supporting the importance of accuracy of the label mass. In this study’s comparison the variation of label mass caused the toxicity to range from minimal to 38% mortality.

The treatment groups dosed with the contaminated MCLR standards did not show consistent differences from the other treatment groups outside of the MCLR concentration variation. TFA represented the largest contaminant ratio as the free acid TFA in Vendor A and F. Although Vendor A contained TFA, the mice treated with the Vendor A MCLR had serum chemistry values similar to the controls most likely due to the lower MCLR concentration. The free acid form (HTFA) is acutely more toxic due to high acidity compared with the sodium salt (NaTFA) [53]. Because TFA can be formed as a metabolite of halothane metabolism, limited toxicological studies of TFA in rodents have been conducted. Blake [54] administered 150 mg/kg of the free acid TFA to mice and 2 of the 5 mice died with no other endpoints reported. In a 1971 study, histologic and electron microscopy sections of livers from mice administered a single i.p. injection of 1000 mg/kg NaTFA 24 h prior demonstrated mild liver changes described as a slight accumulation of lipids and glycogen. Serum chemistry parameters were not performed in this study [55]. Since the exact amount of TFA in the present study was not determined, it is difficult to compare, but given the large exposure in the Blake and Rosenberg studies and lack of toxicity in Vendor A, any contribution from the TFA in the MCLR seems minor.

This study was prompted by poor repeatability of hepatotoxicity endpoints in previous experiments as our lab completed preliminary dose-finding for a MC congener comparative toxicity study. After consulting with chemists familiar with cyanotoxins, we found that the need for standard mass verification was anecdotally known among some professionals but was not formally published. The authors received encouragement from cyanotoxin researchers to make this information published and available. During the review process for this manuscript, The Water Research Foundation published a project report also detailing discrepancies in commercially prepared cyanotoxin standards between vendors and even between lots from the same vendor [43].

The commercial market for cyanotoxin standards, and possibly other natural toxins that cannot be synthesized, seems to lack standardized quantity and purity accountability, and validation is up to the individual vendor. HPLC or HPLC-PDA is often used by vendors to verify purity, but PDA/UV is more susceptible to interference during measurement which may affect the mass assessment. Based on the present study, it is evident that end users of cyanotoxin standards need to be verify quantity and purity with analytical methods although immediate access to a chemist familiar with these procedures is not equally available to all cyanotoxin researchers.

It is important to note that these seven samples represent one point in time and samples from the same seven vendors now or in the future may or may not be the same. Until industry accuracy is more standardized for quantity and purity, label mass variability could continue. These variations may lead to incorrect conclusions about data and consequent failure to replicate reported studies. Interpretation of past studies that do not note source, purity or mass validation of toxins will create ambiguity concerning the data. Lack of vital information supplied with the products such as expiration date, lot number, and COA affects researchers’ ability to follow good quality assurance practices. Methods presented here along with available CRMs can be used to verify the accuracy of purity and quantity for vendor supplied MCLR standards [37,42].

## 4. Methods

### 4.1. Animals

Five to six-week-old CD-1 mice were obtained from Charles River Laboratories (Raleigh, NC, USA). The animals arrived at the Center for Public Health and Environmental Assessment (CPHEA) animal facility and allowed to acclimate for at least 5 days prior to initiation of the experiments. Animals were housed three to a cage in polycarbonate cages on heat-treated pine shaving bedding in animal rooms with a controlled temperature range (20–22 °C) and a 12:12 h light–dark cycle. Animals were fed commercial rodent chow (Purina Prolab) and filtered (5 µm) municipal tap water ad libitum. All studies were conducted after approval of animal research plan #18-09-002 by the Institutional Animal Care and Use Committee (IACUC) using recommendations of the 2011 NRC “Guide for the Care and Use of Laboratory Animals” [56], and the Public Health Service Policy on the Humane Care and Use of Laboratory Animals (2015) [57].

### 4.2. Experimental Design

#### 4.2.1. Microcystin-LR Standards Preparation

Standards of MCLR purchased from seven vendors were shipped to the Environmental Protection Agency’s Office of Research and Development in Research Triangle Park, North Carolina (EPA, NC). MCLR vendor samples were purchased from Enzo Life Sciences, Greenwater Laboratories, Marbionc, Apexbio, Calbiochem, Beagle Bioproducts and Cayman Chemical, but results presented here will remain anonymous. All samples were received dry in glass vials, stored at −20 °C until reconstitution, and analyzed within the expiration dates if available. Vendor B was unintentionally left in shipping package at room temperature for 7 days prior to −20 °C storage. Reconstitution occurred within 30 days of sample receipt and sample analysis occurred within 35 days of reconstitution. All vendor samples were reconstituted in their received vials using HPLC grade water to a nominal concentration of 250 ng/µL. For four vendor samples where 0.1 mg of material or less was provided, a concentration of 100 ng/µL was used instead. For Vendor B, the provided sample vial was too small to contain the target concentration, so the sample was quantitatively transferred to another glass vial with multiple rinses and brought to the target concentration [47].

HPLC grade water was used for sample preparation because these samples were also used for the in vivo study in parallel. To determine if the use of water without methanol impacted solubility, since EPA Method 544 and the Beer-Lambert Law use methanol [37,58,59], two additional vials of MCLR from the same lot were solubilized in either water only or methanol only for comparison. Samples were stored at 4 °C for a week prior to analysis. When the methanol only and water only samples were analyzed in quintuplicate using the MS-TOF methods outlined below, there was no significant difference in the peak area response (*p* value = 0.265, Figure S1) indicating the use of pure water did not affect sample solubility.

#### 4.2.2. Animal Dosing, Observation and Necropsy

A single intraperitoneal (i.p.) injection of MCLR was administered at 40 µg/kg based on the mass purchased from each vendor. The dose of 40 µg/kg i.p. had been determined previously in our lab as a toxic dose level (See Supplemental Figure S1). Each treatment group contained 24 mice divided equally between males and females. Twenty-four hours post dosing, all animals were anesthetized by CO_2_ inhalation, weighed, euthanized by exsanguination (blood collection) and necropsies performed. Blood was obtained transdermally from the heart with a 25-gauge 5/8 in needle attached to a 1 mL syringe. Blood collected in 0.5 mL serum separator tubes was allowed to clot ≥30 min at room temperature and was centrifuged at 13,000× *g* for 90 s to separate the serum which was placed in a separate 1.5 mL microcentrifuge tube and stored at −20 °C until the day of analysis. All necropsies were done experimenter blind. Necropsies were performed immediately after blood collection, and a gross assessment of liver appearance was recorded and then converted to a liver score [10,11]. The liver score was based on presence/severity of lesions and extent of liver surface area affected. Lesions were the visual anomalies of congestion/hemorrhage, infiltration of glycogen or lipid (seen as pale areas), or a reticulated pattern (pronounced pattern of the lobules). The scores given to individual animals ranged from 1–16, and normal was considered a score of 0–2, mild 3–5, moderate 6–8, and severe ≥9 with 16 representing death within the first 12 h. The ranges correlate with severity of toxicity evidenced by clinical signs of moribundity and the serum chemistry results. The score of 16 was added because an earlier death due to toxicity may preclude lesion development and the severe morbidity of death should be reflected in the value. The liver was then removed and weighed, and for comparison, the liver/body weight value was used to normalize the individual liver weight for body size.

#### 4.2.3. Clinical Chemistry

All serum clinical chemistry analyses were performed using the Randox Daytona Plus (Belfast, UK). Due to the small volume of serum obtained, duplicate tests were not run. Hepatic cell injury was assessed by determining the serum activities of alanine aminotransferase (ALT), aspartate aminotransferase (AST), and glutamate dehydrogenase (GLDH). Markers of potential renal injury included serum concentrations of blood urea nitrogen (BUN) and creatinine (Cr). Serum albumin, globulin and total protein were also measured as markers of general health. All assays were performed using reagents obtained from the instrument manufacturer.

### 4.3. MCLR Analysis by UV-Vis at EPA, NC

The seven MCLR vendor samples were quantified by UV absorbance using a Nanodrop ND-1000 Spectrophotometer at 238 nm. Original stock solutions of vendor samples were measured directly without dilution. A HPLC grade water sample was measured as a blank. Concentrations were determined from absorbance using the Beer-Lambert Law and a molar extinction coefficient of 39,000 L/mol cm for MCLR. While this extinction coefficient was developed for MCLR dissolved in 100% MeOH, it is also applicable for MCLR dissolved in HPLC grade water [58,59].

### 4.4. MCLR Analysis by MS-TOF at EPA, NC

Following the reconstitution with HPLC grade water, MCLR samples were vortexed for 1 min and stored at 4 °C until analysis.

The seven MCLR vendor samples were quantified and screened for non-targeted contaminants by LC-MS-TOF using an Agilent 1100 series HPLC equipped with an Eclipse Plus C8 column (2.1 × 50 mm, 3.5 μm; Agilent) interfaced to an Agilent 6210 series Accurate-Mass MS-TOF system with both positive and negative electrospray ionization (ESI). Supplies used to prepare samples for LC-MS-TOF analysis include polypropylene pipette tips and LC vials and polypropylene vial caps lined with polytetrafluoroethylene (PTFE). The 250 ng/µL vendor samples were diluted 10× in HPLC grade water prior to analysis, while samples dissolved at 100 ng/µL were measured directly. Sample aliquots of 20 µL were diluted in the LC vials with 100 µL MeOH, and 280 µL 0.4 mM ammonium formate (20-fold dilution). Five microliters of each diluted sample were injected and separated using a linear gradient. All samples were prepared in triplicate with triplicate injections of each preparation. Sample blanks (i.e., MeOH and HPLC grade water) were analyzed similarly to determine if any impurities originated from the analytical method.

Quantification was carried out using MS peak area abundance in negative mode (− = 993.5415) with a three-point external calibration curve (i.e., 1.3, 6.3, and 12.6 ng on-column) prepared using a CRM purchased from NRC Canada. The CRM was received in 50:50 methanol:water and diluted with variable amounts of deionized (DI) water in the LC vials for analysis. All the standards had final ratios of methanol and water in the LC vials equal to the samples (75:25 water:methanol). The mobile phase system consisted of 0.4 mM ammonium formate in 95:5 DI water:methanol (A) and 95:5 methanol:DI water (B). The initial mobile phase (25% B) was ramped to 80% B over 5 min, then to 100% over 2 min. The mobile phase was held at 100% B for 3 min followed by a 4 min post time at the starting LC conditions. This procedure is a modified EPA Method 544 [37].

Vendor samples run on the MS-TOF were analyzed using a non-targeted screening process to identify potential contaminants using the Agilent Mass Hunter software package. Molecular features, consisting of an accurate mass, chromatographic peak, and mass spectrum, were extracted using Agilent Profinder in recursive feature extraction (small molecule) mode and mutually aligned between all vendor samples and the blanks. Compounds that were present in vendor samples but not present in the blank were manually examined for putative identification.

### 4.5. MCLR Analysis by LC-MS/MS at EPA NV

Brown glass vials with polypropylene PTFE vial caps were used to ship reconstituted, undiluted aliquots of MCLR vendor samples to EPA in Las Vegas, Nevada (EPA, NV) for validation of our standards measurements. Samples were shipped from the EPA, NC to the EPA, NV overnight on ice.

The seven MCLR vendor samples were also quantified by HPLC-MS/MS using an Agilent 1290 Infinity II HPLC coupled to an Agilent 6495 Triple Quadrupole MS with positive ESI. The HPLC was equipped with a BEH phenyl column (2.1 × 100 mm, 1.7 μm, Waters Corp, Milford, MA, USA) at 35 °C and mobile phase running at 300 μL/min consisting of (A) 0.05% acetic acid in 95:5 DI water-methanol and (B) 0.05% acetic acid in 95:5 methanol-DI water. All samples received were diluted accordingly such that two concentrations (5 and 50 pg/μL) from each vendor were used for the analysis. A 20-μL diluted sample was injected and eluted with the following gradient: initial condition of 35% B ramped to 90% B over 5 min and held for 1.5 min, then back to initial condition over 3.5 min followed by a 3 min post time equilibration prior to the next run. The MS acquisition was performed in positive polarity using the ^2+^ precursor ion 498.3 with product ions 135.2, 105.1 and 103.1. An external five-point calibration curve was prepared using NRC Canada CRM and quantified using product ion 135.2 peak area.

### 4.6. Statistical Analysis

All analyses were done using SASv9.4 software. Analyses were done separately by sex. Means and standard errors were calculated by SAS Proc Means. Continuous variables were analyzed with two-way main effects ANOVA using Proc Mixed, with predictors Vendor and Block. The predictor for block was included in the ANOVA model to adjust for differences in means across blocks. The ANOVA assumptions of normality and homogeneity of variance were tested with the Shapiro–Wilk test (Proc Univariate) and Levene’s test (Proc GLM), respectively. ANOVA analysis was calculated using untransformed or log_10_ transformed values, depending on which best met these assumptions for each outcome variable. Pairwise *t*-tests comparing each vendor group with the control were calculated with and without Dunnett’s adjustment for multiple comparisons.

Liver Score (an ordinal variable) was analyzed with Cochran Mantel Haenszel chi-square tests in SAS Proc Freq. The alternative hypothesis that mean scores were equal for all groups was used to test the complete set of vendors, and the difference in mean score for each vendor group vs. the control.

Spearman correlations (Proc Corr) (not shown) were used to examine the relationship between each outcome variable and each calculated actual dose (UV, LC-MS/MS, MS-TOF) for each vendor group, and between each outcome variable and liver score. The *p* values were similar for most endpoints by each method, and the MS-TOF measurement was selected as the dose to represent each vendor for the toxicology calculations. This correlation is based on a ranking of the data, so does not depend on (linear or log) scale.

## Figures and Tables

**Table 1:** Contaminants identified in MCLR vendor samples using time of flight mass spectrometry (MS-TOF).

Suggested Formula	Monoisotopic Mass of Unknown (Da)	Ratio of Unknown Compound to MCLR Peak Area						
		Vendor						
		A	B	C	D	E	F	G
C_2_HF_3_O_2_—TFA ^1^	113.993	11.3					17.8	
SO_4_ Sulfate Cluster	195.936					0.83		
C_48_H_72_N_10_O_12_- d-meth MCLR	980.533		0.02					
C_50_H_76_N_10_O_12_—MCLR-Methyl Ester	1008.564		0.01					

^1^ Trifluoroacetic acid (TFA).

**Table 2:** MCLR Toxicity after 40 µg/kg i.p. (based on vendor label) in the CD-a mouse ^1^.

	Number of Animals		Liver Wt (g)		Liver/Bwt %		Weight Change (g)		Liver Score	
Vendor (µg/kg MCLR)	Male	Female	Male	Female	Male	Female	Male	Female	Male	Female
Control	18	18	2.1 ± 0.1	1.4 ± 0.1	6.1 ± 0.15	5.4 ± 0.14	−0.09 ± 0.08	−0.19 ± 0.16	1.9 ± 0.35	1.2 ± 0.35
A (13.2)	12	12	2.0 ± 0.1	1.4 ± 0.0	5.7 ± 0.19	5.5 ± 0.17	−0.41 ± 0.13	−0.20 ± 0.15	3.0 ± 0.51	2.8 ± 0.52 ^a^
B (25.6)	12	12	2.1 ± 0.1	1.5 ± 0.1	6.0 ± 0.19	5.7 ± 0.17	−0.20 ± 0.14	−0.49 ± 0.11	4.3 ± 0.45 ^c^	2.9 ± 0.34 ^b^
C (34.0)	12	12	2.0 ± 0.1	1.5 ± 0.1	6.0 ± 0.19	5.9 ± 0.17 ^a^	−0.12 ± 0.17	−0.41 ± 0.19	5.7 ± 0.53 ^c^	3.1 ± 0.67 ^a^
D (35.6)	12	12	2.0 ± 0.1	1.5 ± 0.1	6.0 ± 0.20	5.9 ± 0.18 ^a^	−0.44 ± 0.13	−0.68 ± 0.34 ^a^	6.3 ± 0.57 ^c^	4.0 ± 0.62 ^c^
E (39.6)	12	12	1.9 ± 0.1	1.7 ± 0.1 ^c^	5.9 ± 0.19	6.2 ± 0.17 ^c^	−0.17 ± 0.34	−0.65 ± 0.25	7.3 ± 0.66 ^c^	4.5 ± 1.12 ^b^
F (38.4)	12	12	2.3 ± 0.1 ^a^	1.6 ± 0.1	7.0 ± 0.21 ^c^	6.2 ± 0.2 ^c^	−0.18 ± 0.15	−0.27 ± 0.27	5.6 ± 0.97 ^c^	6.6 ± 1.05 ^c^
G (44.8)	12	12	2.2 ± 0.1	2.0 ± 0.1 ^c^	6.6 ± 0.19	7.8 ± 0.20 ^c^	−0.53 ± 0.22	−0.24 ± 0.13	7.6 ± 0.94 ^c^	9.4 ± 0.43 ^c^
	**ALT (IU/L)**		**AST (IU/L)**		**GLDH (IU/L)**		**BUN (mg/dL)**		**Creatinine (mg/dL)**	
**Vendor (µg/kg MCLR)**	**Male**	**Female**	**Male**	**Female**	**Male**	**Female**	**Male**	**Female**	**Male**	**Female**
Control	33.4 ± 2.2	43.4 ± 6.7	69.0 ± 7.7	98.2 ± 10.9	13 ± 1.7	10.7 ± 1.2	11.1 ± 0.22	9.6 ± 0.27	0.48 ± 0.01	0.6 ± 0.06
A (13.2)	42.9 ± 3.9	89.2 ± 40.3	72.5 ± 9.1	142.1 ± 43.7	22.7 ± 5.2	13 ± 2.2	9.6 ± 0.46 ^a^	8.1 ± 0.39 ^a^	0.5 ± 0.00	0.5 ± 0.03 ^a^
B (25.6)	145.9 ± 22.3 ^b^	104.5 ± 27.8 ^b^	124 ± 2.5	140.8 ± 26.4	37.3 ± 8.1 ^a^	27.9 ± 5.0 ^b^	10.7 ± 0.87	9.2 ± 0.50	0.4 ± 0.01 ^a^	0.6 ± 0.05
C (34.0)	820.6 ± 395.1 ^c^	567.3 ± 431.2 ^b^	372.4 ± 218.0 ^a^	535.2 ± 407.7	108.2 ± 29.9 ^b^	28.6 ± 8.1 ^a^	9.9 ± 0.41	8.0 ± 0.40 ^b^	0.5 ± 0.01	0.7 ± 0.09
D (35.6)	743.1 ± 224.5 ^c^	676.5 ± 423.8 ^c^	276 ± 83.5	479.8 ± 283.7 ^a^	152.1 ± 32.6 ^c^	64.3 ± 7.6 ^c^	9.8 ± 0.25 ^a^	9.1 ± 0.34	0.5 ± 00	0.8 ± 0.10
E (39.6)	435.8 ± 166.4 ^c^	750.3 ± 403.7 ^c^	725.9 ± 553.2 ^b^	678.1 ± 441.2 ^b^	78.8 ± 23.8 ^b^	62.3 ± 16.0 ^c^	11.4 ± 1.08	9.1 ± 0.60	0.6 ± 0.08	0.6 ± 0.06
F (38.4)	864.1 ± 314.9 ^c^	715.4 ± 153.5 ^c^	328.1 ± 105.4 ^c^	681.2 ± 341.8 ^c^	No data	198.8 ± 44.3 ^c^	11.7 ± 0.67	9.6 ± 0.42	0.5 ± 0.05	0.4 ± 0.03 ^a^
G (44.8)	2467.2 ± 1089.4 ^c^	2997.4 ± 806.8 ^c^	962.6 ± 403.8 ^c^	2191.1 ± 1140.6 ^c^	81.1 ± 31.4 ^b^	72 ± 23.0 ^c^	10.3 ± 0.88	15.3 ± 1.76 ^c^	0.5 ± 0.02	0.8 ± 0.10
	**Albumin (g/dL)**		**Globulin (g/dL)**		**Total Protein (g/dL)**		**Early Deaths**			
**Vendor (µg/kg MCLR)**	**Male**	**Female**	**Male**	**Female**	**Male**	**Female**	**Male**	**Female**		
Control	3.1 ± 0.04	3.6 ± 0.10	2.1 ± 0.03	2.1 ± 0.06	5.2 ± 0.06	5.3 ± 0.13	0	0		
A (13.2)	3.1 ± 0.05	3.6 ± 0.14	2.2 ± 0.04	2.0 ± 0.05	5.2 ± 0.07	5.0 ± 0.18	0	0		
B (25.6)	3.1 ± 0.06	3.8 ± 0.12	2.2 ± 0.11	2.2 ± 0.04 ^a^	5.2 ± 0.11	5.4 ± 0.19	0	0		
C (34.0)	3.1 ± 0.05	3.7 ± 0.14	2.3 ± 0.03 ^b^	2.2 ± 0.07	5.4 ± 0.07	5.1 ± 0.19	0	0		
D (35.6)	3.1 ± 0.06	3.6 ± 0.11	2.3 ± 0.04	2.5 ± 0.10 ^b^	5.3 ± 0.05	5.8 ± 0.10	0	0		
E (39.6)	3.2 ± 0.05 ^a^	3.4 ± 0.12	2.3 ± 0.03 ^b^	1.9 ± 0.11	5.1 ± 0.17	5.0 ± 0.17	1	2		
F (38.4)	2.9 ± 0.06 ^a^	3.5 ± 0.11	2.2 ± 0.08	2.3 ± 0.12	5.4 ± 0.11 ^a^	5.5 ± 0.16	3	1		
G (44.8)	3.0 ± 0.06	3.1 ± 0.11 ^c^	2.2 ± 0.06 ^a^	1.7 ± 0.14 ^b^	5.0 ± 0.15	4.8 ± 0.33	2	7		

^1^ All significance tests of difference from control. Mortality statistical significance by Fisher’s exact test; liver score by Cochran–Mantel–Haenszel chi-square; all other endpoints by ANOVA/*t*-test. MCLR dose is based on measurement by MS-TOF. ^a^ = *p* ≤ 0.05 for differences from controls. ^b^ = *p* ≤ 0.01 for differences from controls. ^c^ *= p* ≤ 0.001 for differences from controls.

## Data Availability

Not applicable.

## Supplementary Materials

The following supporting information can be downloaded at: https://www.mdpi.com/article/10.3390/toxins14100705/s1, Figure S1: Data graphed from the MCLR dose-finding study done to determine intraperitoneal (i.p.) dose for vendor comparison study. N = 3 male and 3 female 5–6 week-old CD-1 mice per dose level of microcystin-LR (MCLR); Table S1: Comparing Pre-Use Standard Information as Provided by Vendor.
