# Supplementary material for: Variability of Microcystin-LR Standards Available from Seven Commercial Vendors
Source: Toxins (Basel). 2022 Oct 14;14(10):705. doi: 10.3390/toxins14100705 (PMC9611723; doi:10.3390/toxins14100705)
Supplement: Supplementary file 1 [file toxins-14-00705-s001.zip › Supplemental Figure S1.pdf]

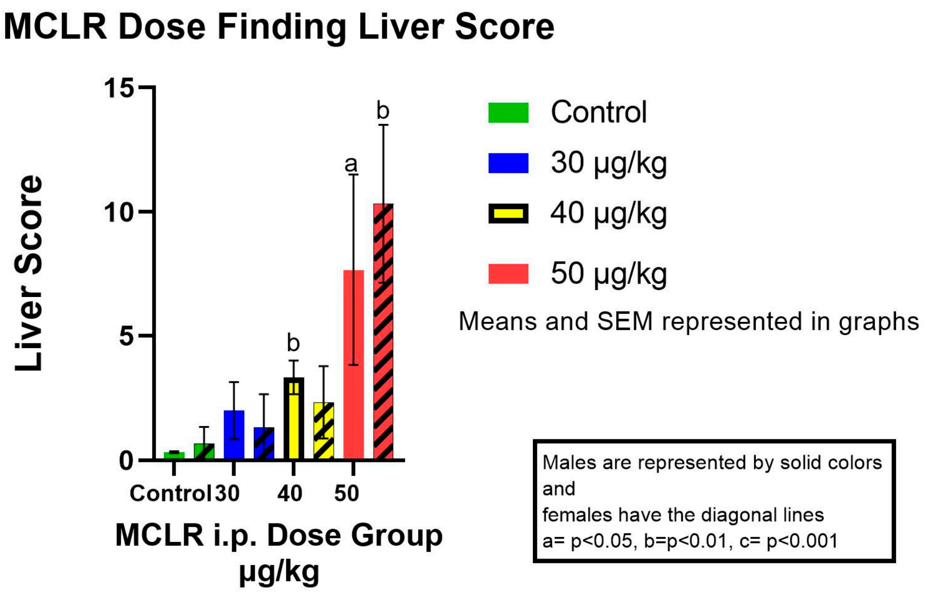
MCLR Dose Finding Liver Score
Liver Score
15
10
5
0
b
a
b
Control
30
40
50
MCLR i.p. Dose Group
µg/kg
Control
30 µg/kg
40 µg/kg
50 µg/kg
Means and SEM represented in graphs
Males are represented by solid colors and
females have the diagonal lines
a= p<0.05, b=p<0.01, c= p<0.001

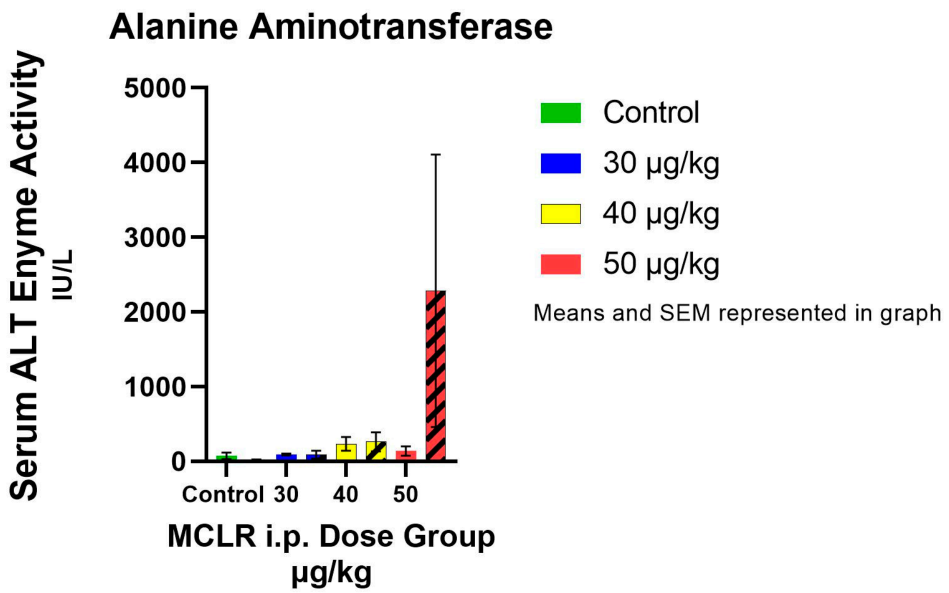
Alanine Aminotransferase
Serum ALT Enyme Activity
IU/L
5000
4000
3000
2000
1000
0
Control
30
40
50
MCLR i.p. Dose Group
µg/kg
Control
30 µg/kg
40 µg/kg
50 µg/kg
Means and SEM represented in graph

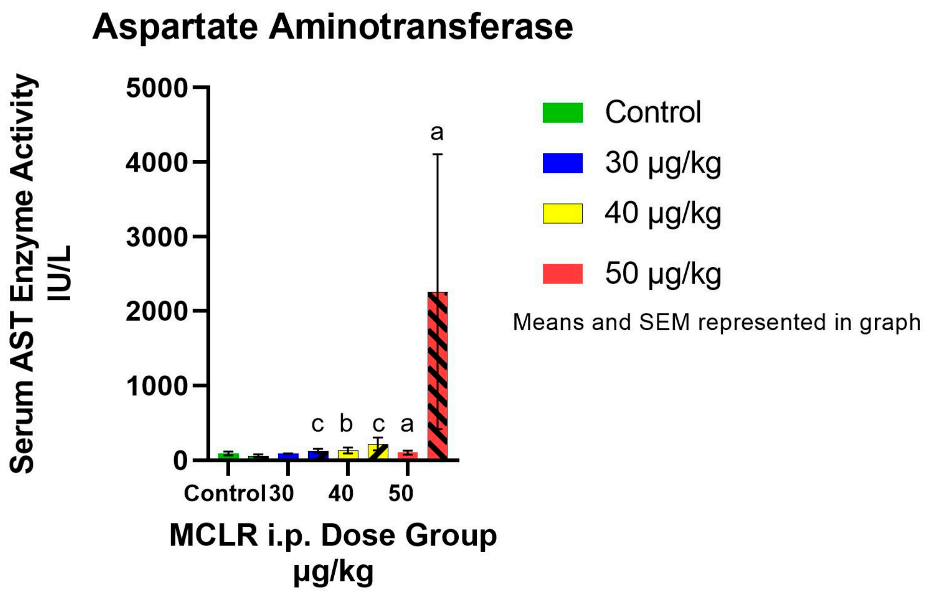

Supplemental Figure S1. Data graphed from the MCLR dose-finding study done to determine intraperitoneal (i.p.) dose for vendor comparison study. N=3 male and 3 female 5-6 week-old CD-1 mice per dose level of microcystin-LR (MCLR).
