# Supplementary material for: Variability of Microcystin-LR Standards Available from Seven Commercial Vendors
Source: Toxins (Basel). 2022 Oct 14;14(10):705. doi: 10.3390/toxins14100705 (PMC9611723; doi:10.3390/toxins14100705)
Supplement: Supplementary file 1 [file toxins-14-00705-s001.zip › Supplemental Table S1.pdf]

Supplemental Table S1. Comparing Pre-Use Standard Information as Provided by Vendor.

Vendor	Date Rec'd	Date water added	COA	µg size purchased	Price	Price per mg	Lot #	Purity	Graph	Stability/ Exp date	Storage
A	12-14-16	12-20-16	MSDS	100	50 +EPA UPS account	500	none	none	N	none	-20C
B	1-3-17-sat at room temp 12-23-16 to 1-3-17	1-13-17	NA online, requested through cs, then Y	500	239 +33 shipping	478	2697030	HPLC 98.2% on label; 98.6% on COA	N	none	-20C
C	11-18-16	12-20-16	Y	500	208 +25 shipping	416	Batch 1	HPLC >98%	N	none	-20C
D	1-20-17	12-12-16	Y	100	44 +23 shipping	440	MCLR-2002	HPLC-PDA ≥95%	Y	≥1 year	-20C
E	11-14-16	12-12-16	Product information	100	45 +34 shipping	450	Batch 0477010-8	≥95%	N	1 year from QC date on product when stored properly; 9-22-17 exp. Date on packing slip	-20C; do not recommend aqueous storage > 1 day
F	1-10-17	1-13-17	Y	500	200	400	5	>95%	Y	≤1 year	-20C
G	11-14-16	12-12-16	Y	100	67 +24 shipping	670	L30360	HPLC>95%	N	none	-20C

Abbreviations used: COA=certificate of analysis, exp=expiration, mg=milligram, rec'd=received, cs=customer service, Y=yes, N=no
